# Albumin-Based Nanocarriers for the Simultaneous Delivery of Antioxidant Gene and Phytochemical to Combat Oxidative Stress

**DOI:** 10.3389/fcell.2022.846175

**Published:** 2022-08-12

**Authors:** Saba Naqvi, Vitaly A. Khanadeev, Boris N. Khlebtsov, Nikolai G. Khlebtsov, Monika S Deore, Gopinath Packirisamy

**Affiliations:** ^1^ Department of Regulatory Toxicology/Pharmacology and Toxicology, National Institute of Pharmaceutical Education and Research, Raebareli, India; ^2^ Nanobiotechnology Laboratory, Department of Biosciences and Bioengineering, Joint Faculty in Centre for Nanotechnology, Indian Institute of Technology Roorkee, Roorkee, India; ^3^ Institute of Biochemistry and Physiology of Plants and Microorganisms, Russian Academy of Sciences, Saratov, Russia; ^4^ Saratov State Agrarian University, Saratov, Russia; ^5^ Saratov State University, Saratov, Russia; ^6^ Department of Biosciences and Bioengineering, Indian Institute of Technology Roorkee, Roorkee, India

**Keywords:** human serum albumin (HSA), sulforaphane, pSOD1 plasmid, L-132 cells, antioxidant activity

## Abstract

Human serum albumin (HSA) nanoparticles are promising biocompatible, nontoxic, and non-immunogenic platforms for biomedical applications such as bioimaging and drug and gene delivery. The development of nonviral gene delivery vectors is a great challenge for efficient and safe gene therapy. Sulforaphane (SF) can stimulate the expression of antioxidant genes *via* activation of a nuclear transcription factor, the erythroid-2 related factor 2 (Nrf-2). Here, we use polyethyleneimine (PEI)-stabilized HSA nanoparticles to stimulate endogenous antioxidant defense mechanisms in lung epithelial cells L-132 through the combinatorial effect of SF drug and antioxidant superoxide dismutase 1 gene (pSOD1 plasmid) delivered by HSA-PEI-SF-pSOD1 nanocomposites (NCs). The developed NCs demonstrated high biocompatibility (L-132 viability, >95%, MTT assay) and high antioxidant activity because of efficient entry of the SOD1 gene and SF-loaded NCs at a very low (3 μg) dose in L-132 cells. A high transfection efficiency of L-132 cells (∼66%, fluorescent microscopy) was obtained with the GFP-tagged transgene SOD1-GFP. We speculate that the antioxidant activity of HSA-PEI-SF-pSOD1 NCs in L-132 cells is due to the initial release of SF followed by subsequent SOD1 gene expression after three to four days of incubation. Hence, the developed HSA-based NCs can be efficient biocompatible nanocarriers for safe and effective drug and gene delivery applications to treat diseases with high oxidative stress due to combinatorial SF and SOD1 gene mechanisms.

## 1 Introduction

Multifunctional human serum albumin (HSA) nanoparticles are a potential candidate in the widespread application of medicine, imaging, and drug delivery, as well as in gene delivery, due to their indispensable nontoxic and nonviral unique vector properties (Zamanian-Azodi et al., 2013; Farjadian et al., 2019). Nanocomposites consisting of human serum albumin seem to be an ideal candidate for the drug delivery system. HSA-based systems have characteristic properties of biodegradability, nontoxicity, and non-immunogenicity and further favored uptake in inflamed tumor tissues (Rubino et al., 1993; Wartlick et al., 2004; Watcharin et al., 2014). Charged groups present in protein further aid in the physical entrapment of encapsulated drugs. Proteins used in nanoformulations include fibroins, albumin, gelatin, gladine, legumin, 30Kc19, lipoprotein, and ferritin proteins. However, albumin has no limitations or disadvantages over other proteins. Albumin is a natural biomolecule that acts as a component for many epithelial and endothelial cells as it is a crucial protein in blood and tissue fluid. It is preferred over synthetic polymers because of its safety, biocompatibility, and biodegradability (Hong et al., 2020). Albumin is the abundantly present plasma protein that comprises more than 50% of the total human plasma proteins having a molecular weight of 66.5 kDa (Bhushan et al., 2015). It is stable for pH ranging from 4–9 and thermostable at 60°C temperature for up to 10 h, thus enabling perfect possibilities for drug delivery in physiological and cancer cell microenvironments. Various albumin-based formulations are in the clinical trial phase such as MTX-HSA, Aldoxorubicin, CJC-1134, Abraxane®, and ABI-008, 99mTc-Albures (Larsen et al., 2016; Bayat Mokhtari et al., 2018), and a few are already approved by the FDA, such as Abraxane, i.e., paclitaxel–albumin nanoparticles, for the treatment of advanced non-small-cell lung cancer and metastatic breast cancer (Gradishar et al., 2005). The unique ligand-delivery characteristics of HSA enable them for higher solubility of HSA conjugated with hydrophobic drugs in blood plasma. Owing to its high charge to mass ratio, albumin easily binds with water, Na^+^, Ca^2+^, K^+^, fatty acids, bilirubin, hormones, and many xenobiotic drugs (Scott, 1988). It helps in improving the pharmacokinetic property of drug molecules in the biological environment. HSA-based nanoplatforms can also be employed in the gene delivery approach. The current gene therapy strategies are restricted by several serious shortcomings such as carcinogenicity, immunogenicity, and inflammation or high-cost production (Teramoto et al., 1995; Check, 2002; Gordon 2002; Sun et al., 2003; Boulaiz et al., 2005; Zaiss and Muruve, 2005). Nonviral vectors proved to be ideal gene transfection carriers as they can overcome hurdles encountered by inefficient gene transfection. Ideally, nonviral vectors should shield the transgene against cytosolic degradation, least or no cytotoxicity, facilitate cell entry of the plasmid due to biodegradable, biogenic in nature, escape endo/lysosome entrapment, and enable the nuclear targeting of the transgene (Luo and Saltzman, 2000). DNA vaccination or gene therapy required transfecting a cell with DNA. DNA viral vectors used for gene delivery are at risk of immune and inflammatory responses (Bessis et al., 2004). Furthermore, nonviral vectors can release plasmid DNA over a prolonged period without any immunogenic and toxic adverse effects and maintain stable gene expression.

Antioxidant and anticancer drug molecule SF has the potential to stimulate the expression of antioxidant genes via activation of nuclear factor erythroid-2 related factor 2 (Nrf-2), a transcription factor that plays a key role in oxidative stress by activating antioxidant enzymes (Nandini et al., 2020). An antioxidant enzyme, superoxide dismutase (SOD1), decreases free oxygen radicals by converting them into hydrogen peroxide, which is further broken down into the water molecule with the assistance of enzymes such as catalase or glutathione peroxidase (Mo et al., 2007). Here, we employ the combinatorial effect of SF drug and antioxidant superoxide dismutase 1 gene (pSOD1 plasmid) encapsulated in polyethyleneimine (PEI) stabilized HSA-based nanocomposites HSA-PEI-SF-pSOD1 to stimulate endogenous antioxidant defense mechanisms in lung epithelial cells L-132. Oxidative stress has a major role in various lung pathologies, including fibrosis and lung epithelial cell apoptosis (Kasper et al., 2000). Oxidative stress pathology involves both free radicals and reactive oxygen species (ROS). However, particularly, superoxide anion and hydroxyl radical contribute mostly to the pathogenesis of various diseases. As superoxide further reacts with nitric oxide, which further contributes to oxidative stress by producing radicals such as peroxynitrite. Earlier studies have shown that decreased oxidative stress decreases the incidence of lung cancer (Ho et al., 2007). Thus, the present study focuses on the development of an albumin-based nanocarrier for the simultaneous delivery of antioxidant genes and phytochemicals to combat oxidative stress.

## 2 Methodology

### 2.1 Materials and Methods

All chemicals were obtained commercially and used without additional purification. Human serum albumin (HSA), glutaraldehyde (GA), D,L-Sulforaphane (≥90%, HPLC), polyethyleneimine (PEI) with average M_w_ = 25,000 (all from Sigma-Aldrich), plasmid pF141 pAcGFP1 SOD1WT (Addgene, https://www.addgene.org/26402/), absolute ethanol (ET0016, 99.99%; Scharlau Chemie, Spain), phosphate buffer saline (PBS) and dimethyl sulfoxide (DMSO) (Reachim, Russia), and MilliQ water (18 mΩ×cm; Millipore). 3-(4,5-dimethylthiazol-2-yl)-2,5-diphenyl tetrazolium bromide (MTT), Dulbecco’s Modified Eagle Medium (DMEM), and the 2',7'- dichlorofluorescein diacetate (DCFH-DA) dye. Commercial SOD assay kits were ordered from Sigma Aldrich. Hydrogen peroxide (30%) was purchased from Merck. Other chemicals used in the current study were of analytical and molecular biology grades. L-132 (Human Lung epithelial cells) cell line was obtained from National Centre for Cell Science, Pune, India. They were maintained in Dulbecco’s modified Eagle’s medium (DMEM) supplemented with 10% calf serum and 1% Penicillin-streptomycin maintained in a 37°C incubator with 5% CO_2_ and 95% air.

### 2.2 Synthesis of HSA-PEI-SF-pSOD1 Nanoparticles

**FIGURE 1:**
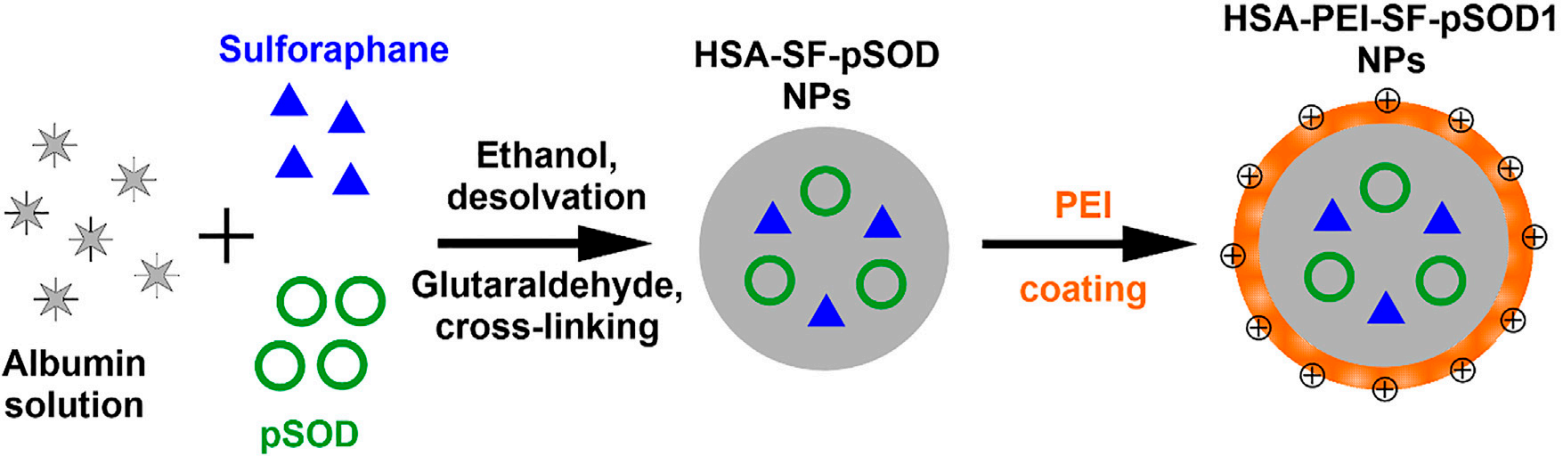
Schematic representation of the basic steps in the fabrication of composite HSA-PEI-SF-pSOD1 nanoparticles.

The scheme of synthesis of HSA-PEI-SF-pSOD1 nanoparticles is presented in Figure 1. It is a coacervation procedure detailed in the study (Galisteo-González and Molina-Bolívar, 2014). Briefly, 100 mg of HSA was dissolved in 10 ml of water followed by the addition of 10 μL of sulforaphane solution in DMSO (100 mg/ml) and 100 μL of plasmid DNA solution (1490 ng/µL). Protein was transformed into NPs in a dropwise manner with the addition of 9.3 ml of absolute ethanol under strong stirring with the help of a magnetic stirrer. The solution turns into white color due to turbidity. To initiate cross-linking of NPs, after 5 min 150 μL of 8% glutaraldehyde water solution was added to the reaction mixture. Stirring of the mixture was carried out overnight at room temperature. During synthesis, color of the solution changes from white to light yellow, indicating the formation of cross-linked protein-based NPs. To eliminate the unreacted substances, the as-prepared HSA-SF-pSOD1 nanoparticles were washed by two-fold centrifugation at 15,000 g for 10 min with redispersion in water. By measuring the extinction of the supernatant after the first washing at a wavelength of 260 nm we found that no differences were observed for the HSA nanoparticles with DNA and without DNA. Thus, all added DNA was loaded into the HSA nanoparticles. To improve the colloid stability, nanoparticles were covered with a layer of polyethyleneimine (PEI) by the addition of PEI water solution to a final concentration of 1 mg/ml. After 24 h incubation under magnetic stirring, the resulting HSA-PEI-SF-pSOD1 NPs were washed by two-fold centrifugation at 15000 g for 10 min and redispersed in 10 ml of water. Unloaded HSA NPs and nanoparticles without SF or plasmid DNA were synthesized by a similar protocol.

### 2.3 Cell Cytotoxicity Evaluation

The biocompatibility of NPs was determined by the cell viability assay, tetrazolium salt-based assays, and 3-(4,5-dimethylthiazol-2-yl)-2,5-diphenyltetrazolium bromide MTT assay. Briefly, L-132 cells were plated at a density of 1×10^4^ cells per well into 96-well plates until subconfluent (Corning, Costar, NY). Twenty four hours after seeding the cells, the cells were then incubated with different (low to high) concentrations (0.2, 0.6, 1.0, 1.5, 2.0, 2.5, 3.0 µg) of the blank HSA-PEI Np’s, HSA-PEI-SF Np’s, HSA-PEI-pSOD1 Np’s, and HSA-PEI-SF-pSOD1 Np’s up to 96 h. Sterile PBS wash was given to cells after 48 h incubation of nanoparticles, followed by the addition of fresh 90 µL DMEM media per well to the cells. 10 µL of MTT (Sigma-Aldrich, United States) reagent (5 mg/ml stock) was added per well, including controls, and incubated at 37°C for 3–4 h incubation. Cells were examined for the appearance of an intracellular punctate purple precipitate under an inverted microscope. Then the medium was discarded from each well and dimethyl sulfoxide (DMSO) i.e. 100 μL was added including controls further after the appearance of purple color precipitate to dissolve the formazan crystals produced. The plate was incubated in the dark for 20–30 min. Measured the absorbance of each well, including blank with the help of a multimode reader (Cytation3, Biotek) at 570 nm and the background control at 690 nm. The untreated cells were considered 100% viable, all the data were performed in triplicate.

Cell viability (%) was calculated as

$$\text{Cell}\;\text{viability}={\left({\text{A}}_{570}{-\text{A}}_{690}\right)}_{\text{treated}}/{\left({\text{A}}_{570}{-\text{A}}_{690}\right)}_{\text{control}}\text{×}100\left(\text{\%}\right),$$

where A_570_ and A_690_ are the absorbances at 570 and 690 nm, respectively.

### 2.4 Superoxide Dismutase 1 Activity Evaluation

The superoxide anion scavenging activity of blank HSA-PEI NP’s, HSA-PEI-pSOD1 Np’s, and HSA-PEI-SF-pSOD1 Np’s at different time intervals (10, 24, and 48 h) were evaluated with a commercially available SOD assay kit (Sigma Aldrich). Briefly, a sample solution (20 μL, i.e., 2 µg SF and 300 ng pSOD1) was added with 200 μL of WST-1 (2-(4-Iodophenyl)-3-(4-nitrophenyl)-5-(2,4-disulfophenyl)- 2H-tetrazolium, monosodium salt) working solution in a 96 well plate. With the addition of 20 μL enzyme (xanthine oxidase) solution, the reaction was initiated and preceded at 37°C for 20 min. Finally, the inhibition activity was measured by taking the absorbance at 450 nm as compared to the control using a Cytation 3 cell imaging multi-mode plate reader (Biotek).

### 2.5 *In Vitro Transfection Studies*

L-132 cells were seeded onto a 24-well plate at a density of 1×10^4^ cells/well 24 h before the transfection experiment. Transfection studies were started when cells reached 70–80% confluent by washing the L-132 cells twice with 1 ml of sterile PBS which was further incubated at 37°C in a serum-free culture medium. Nanoformulations were added into the well as follows: 1) blank HSA-PEI Np’s in serum-free medium at the same concentrations as in (2); 2) HSA-PEI-SF-pSOD1 Np’s containing 30 µL (460 ng) of plasmid DNA in serum-free medium, 3) 5000 ng of free pSOD1 in a serum-free medium; 4) 400 µL of serum-free culture medium as negative control. Treatments were terminated after a 6 h incubation and the cells were maintained in a complete medium for another 48 h. After incubation with nanoformulations and subsequent washing, the cells were visualized. The intracellular gene expression in each well was reflected by the fluorescent intensity of the images and that was examined by the EVOS cell imaging system (Life Technologies, United States).

### 2.6 Cellular ROS Detection Assay

L-132 cells were plated into six-well plates and grown to optimal confluence. Subsequently, the cells were incubated with 30 µL of HSA-PEI-SF-pSOD1 Np’s (460 ng pSOD1 & 3 µg SF) and blank HSA-PEI Np’s for different periods i.e. 12, 24, and 96 h. After incubation, the cells were washed 3 times with PBS followed by exposure to 200 μM H_2_O_2_ in a culture medium for 4 h at 37°C. The 2’,7'- dichlorofluorescein diacetate (DCFH-DA, Sigma Aldrich) dye was used to measure intracellular ROS production. Inside the cell, deacetylation of DCFH-DA into nonfluorescent 2’,7'-dichlorofluorescein (DCFH) is carried out by endogenous esterases, which is further transformed into dichlorofluorescein (DCF) compound that produces green fluorescence in response to ROS generation that was analyzed by fluorescence microscopy. The stock solution of DCFH-DA was prepared in dimethyl sulfoxide (DMSO, Sigma Aldrich). After the treatment, the cells were given a thorough PBS wash, followed by incubation with 20 μM DCFH-DA at 37°C for 30 min. At the end of incubation, the cells were thoroughly washed; the intracellular antioxidant activity of HSA-PEI-SF-pSOD1 Np’s was studied by the EVOS cell imaging system (Life technologies, United States). The intracellular ROS level in each sample was reflected by the green fluorescent intensity of the cells.

### 2.7 Statistical Analysis

Data are presented as mean ± standard error of the mean and were analyzed by using Microsoft Excel and the GraphPad Prism 5 software (GraphPad, v. 6.1). Statistical analyses were performed using Student’s *t*-test for unpaired data or by two-way ANOVA whichever was applicable and *p <*0.05 was considered statistically significant.

## 3 Result and Discussion

### 3.1 Properties of HSA-PEI-SF-pSOD1 Nanoparticles

**FIGURE 2:**
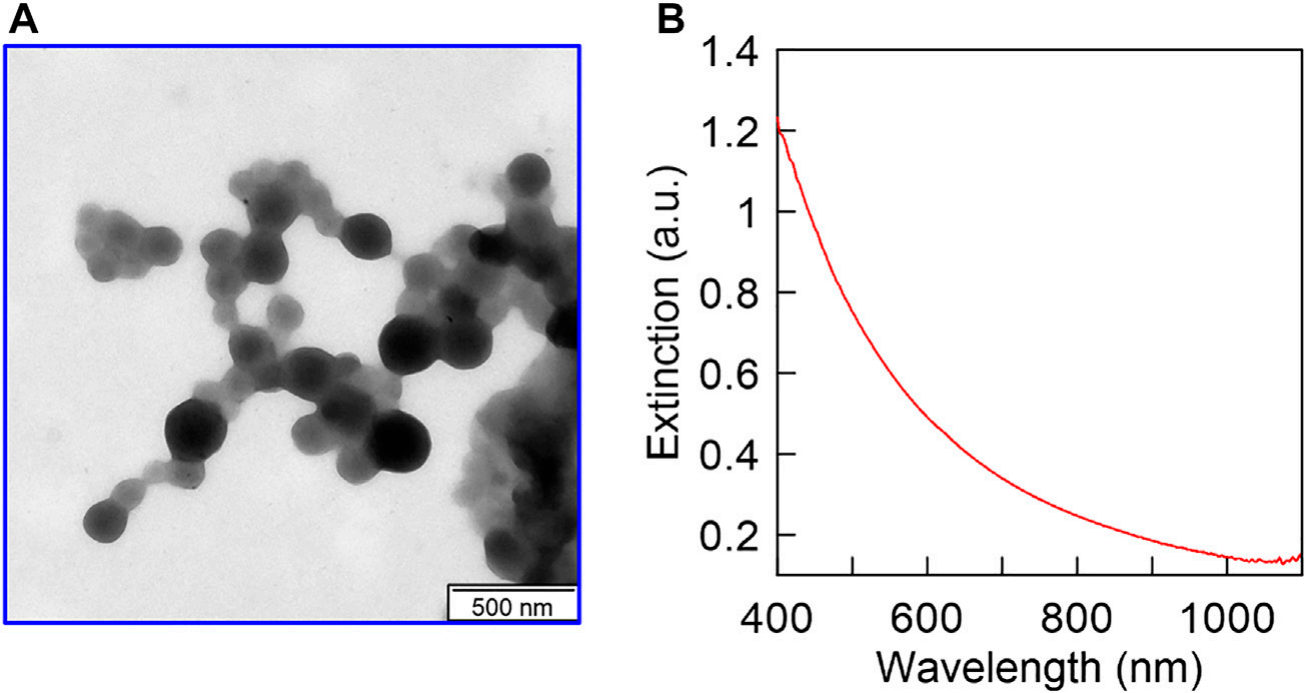
The TEM image **(A)** and extinction spectrum **(B)** of the synthesized HSA nanoparticles.

The average diameters of the synthesized HSA nanoparticles and their polydispersity indices were measured using the dynamic light scattering method and are included in Table 1. A TEM image of unloaded HSA nanoparticles is presented in Figure 2A. An important feature of our synthesis protocol is the fact that loading with the target substance occurs during the synthesis of nanoparticles. In the synthesis of other nanocontainers, such as mesoporous silica, nanoparticle synthesis, and drug loading are usually two distinct steps. In our synthesis, on the contrary, there is a simultaneous synthesis of nanoparticles and loading with the target substance. Therefore, the final size of nanoparticles differs depending on what substances were loaded into them during the synthesis (Table 1). The extinction spectrum of the synthesized unloaded HSA nanoparticles is shown in Figure 2B. The nanoparticle loading with sulforaphane and plasmid DNA as well as PEI coating did not cause noticeable changes in their extinction spectrum (data not shown). The extinction spectrum is typical for non-absorbing particles, which scatter light. This is because sulforaphane, plasmid pSOD1, and PEI do not show significant interaction with light from this wavelength range (from 350 to 1100 nm). The PEI coating of the HSA nanoparticles led to a change of the zeta potential of the nanoparticles from negative (for uncoated HSA particles) to positive (Table 1) to enhance the colloidal stability of particles. In addition, the PEI coating noticeably increases the hydrodynamic radius of the nanoparticles, which is shown in Table 1. After the PEI coating, the particle size always increases. However, the data of DLS measurements convincingly indicate that there is no aggregation of nanoparticles in this case. The point is that the relatively small value of PdI is still preserved, which indicates the absence of aggregation. HSA is conjugated with polyethyleneimine (PEI) to improve the effect of PEI on gene transfection (Baker et al., 1997). The positive surface charge due to the PEI coating helps in efficient cell targeting.

**TABLE 1:** Average DLS diameter, polydispersity index (PdI), and zeta potential of the HSA nanoparticles.

Sample	Average diameter (nm)	PdI	Zeta potential (mV)
HSA NPs	174.5	0.144	−6.4
HSA-PEI NPs	267.5	0.081	59.5
HSA-SF NPs	494.4	0.213	−16.2
HSA-PEI-SF NPs	522.1	0.176	57.4
HSA-pSOD1 NPs	385.4	0.161	−15.3
HSA-PEI-pSOD1 NPs	468.2	0.134	50.9
HSA-SF-pSOD1 NPs	521.8	0.098	−18.2
HSA-PEI-SF-pSOD1 NPs	668.6	0.225	53.8

Surface characteristics, physical stability, and particle size play a very vital role in nano-drug delivery (Langer et al., 2003). As particle size and zeta potential of the nanoparticles ensure maximum efficacy to reach the target site. The size ranges from 10 to 100 nm easily get entry into the systemic circulation and undergo clearance (Longmire et al., 2008), whereas the size ranging from 250 to 1000 nm are encountered phagocytosis and taken up by macrophages a subsequently untaken by the reticuloendothelial system (RES). Through the process of opsonization, macrophage removes the nanoparticles/particles from blood circulation (Harashima et al., 1994; Longmire et al., 2008). A lower surface curvature of the nanomaterials also decreases the possibility of opsonization (Owens and Peppas, 2006).

### 3.2 Cell Viability Assay

The MTT assay demonstrates the cytotoxicity which is based on the conversion of MTT dye to insoluble formazan, which was further detected by spectrophotometry (Li et al., 2009). The cytotoxicity of blank HSA-PEI NPs, HSA-PEI-SF Np’s, HSA-PEI-pSOD1 Np’s, and HSA-PEI-SF-pSOD1 NPs was determined by incubating L-132 cells at concentrations ranging from 2-3.0 µg for up to 96 h. The viability of HSA Np’s-treated cells was examined using the MTT assay. The results demonstrated that blank HSA-PEI Np’s, HSA-PEI-SF Np’s, HSA-PEI-pSOD1 Np’s, and HSA-PEI-SF-pSOD1 Np’s were found to be nontoxic to L-132 cells up to a concentration of 30 µL (i.e., 3.0 µg- SF).

**FIGURE 3:**
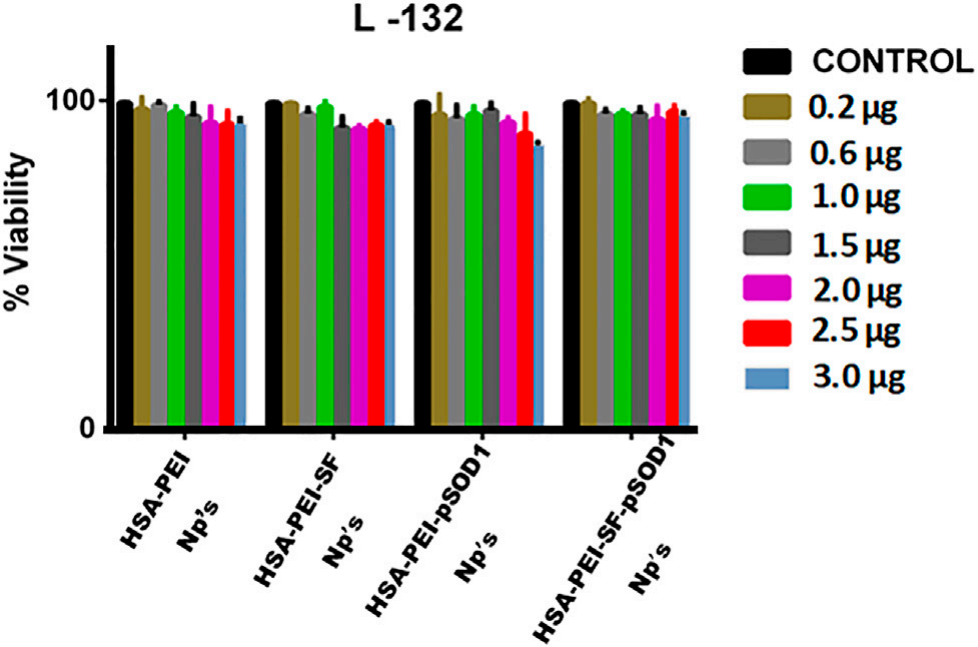
The effects of blank HSA-PEI Np’s and loaded with Sulforaphane and pSOD-1 transgene on cell proliferation and viability of L-132 cells as determined by the MTT assay. Concentration-dependent cytotoxic effects of nanoparticles were evaluated after 96 h incubation. Results are represented as mean ± SD. *Significant difference from control (*p <*0.05).

As shown in Figure 3, the percentage of the viable cell was more than 90% up to 96 h at nanoparticle concentrations up to 3.0 µg. This confirms that the HSA nanocomposites are safe in nature.

### 3.3 SOD Activity of HSA Np’s Loaded With the SOD1 Gene and Antioxidant Drug Sulforaphane

The short-lived radical superoxide anion (O_2_^−^) is produced by an electron addition to oxygen. When environmental determinants such as UV light, environmental pollutants, cigarette smoke, and *γ*-radiation or oxidases such as NADPH oxidase or xanthine oxidase are exposed to cells, these free radicals such as superoxide anion (O_2_^−^) are formed. Once formed, O_2_^−^ attacks the cellular components and leads to damage to the cell membrane, lipids, proteins, and DNA. Endogenously ROS is produced due to metabolic reactions taking place in a body or response to external factors. In the respiratory tract, it is produced through the activation of phagocytes (Ho et al., 2001). ROS is important for the initiation and promotion of cells to neoplastic growth (Ho et al., 2001).

The damage caused by free radicals leads to the development of certain diseases, including cancer, rheumatoid arthritis, atherosclerosis, diabetes, liver damage, and central nervous system disorders.

**FIGURE 4:**
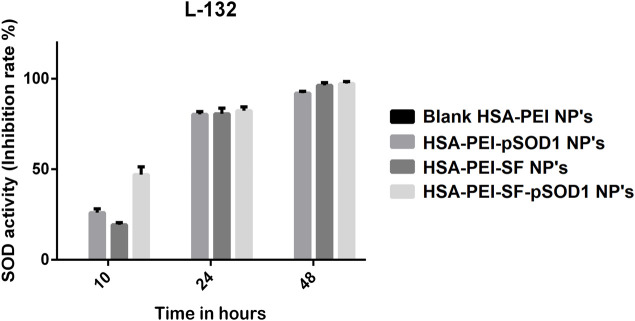
The SOD activity (inhibition rate %) of the blank HSA-PEI Np’s and loaded with Sulforaphane and pSOD-1 transgene showing an elevation in SOD activity as a function of time.

The SOD mimetic activity of blank HSA-PEI Np’s, HSA-PEI-SF Np’s, HSA-PEI-pSOD1 Np’s, and HSA-PEI-SF-pSOD1 Np’s was studied in L-132 cell line against H_2_O_2_ mediated oxidative stress for 6-hour incubation were shown in Figure 4. We observed an elevation in the SOD mimetic activity which is time-dependent on HSA-PEI-pSOD1 Np’s and HSA-PEI-SF-pSOD1 Np’s, which correlates with the enhanced concentration of antioxidants with dual loading of the SOD1 gene and antioxidant sulforaphane drug in the HSA Np’s, which further promotes an increased level of antioxidants in the surrounding cytosolic medium which is when released from the core of HSA Np’s. During the first 10 h, due to the initial burst release of SOD1 and drug from HSA Np’s, a significant increment in the SOD activity was observed as an effect of the higher concentration of expression of gene SOD1 and drug in the surrounding medium. On the other hand, we did not notice any antioxidant activity in blank HSA Np’s, which further confirms the expression of the SOD1 gene and sulforaphane drug. In our results, we observed that HSA Np’s loaded with SOD1 gene and sulforaphane showed higher antioxidant activity at 48 h as compared to the initial 10 h of incubation, this is maybe due to subsequent expression of SOD1 gene in cells after 48 h. After 24 and 48 h, the antioxidant activity of the three types of loaded nanoparticles is approximately at the same level, the so-called saturation effect is observed at a level of about 80 and 100%, respectively. During reaction time, free radicals were generated due to the enzymatic activity of xanthine oxidase, which further converts WST-1 working solution into end product i.e., water-soluble colored formazan dye, which exhibits maximum absorbance at 450 nm. The addition of HSA-PEI-pSOD1 Np’s and HSA-PEI-SF-pSOD1 Np’s inhibits the color formation by actively removing the superoxide radicals that lead to the reduction in the absorbance value (Singh et al., 2010; Bhushan and Gopinath, 2015).

### 3.4 Human Serum Albumin Nanoparticles Facilitate Gene Expression *In Vitro*

**FIGURE 5:**
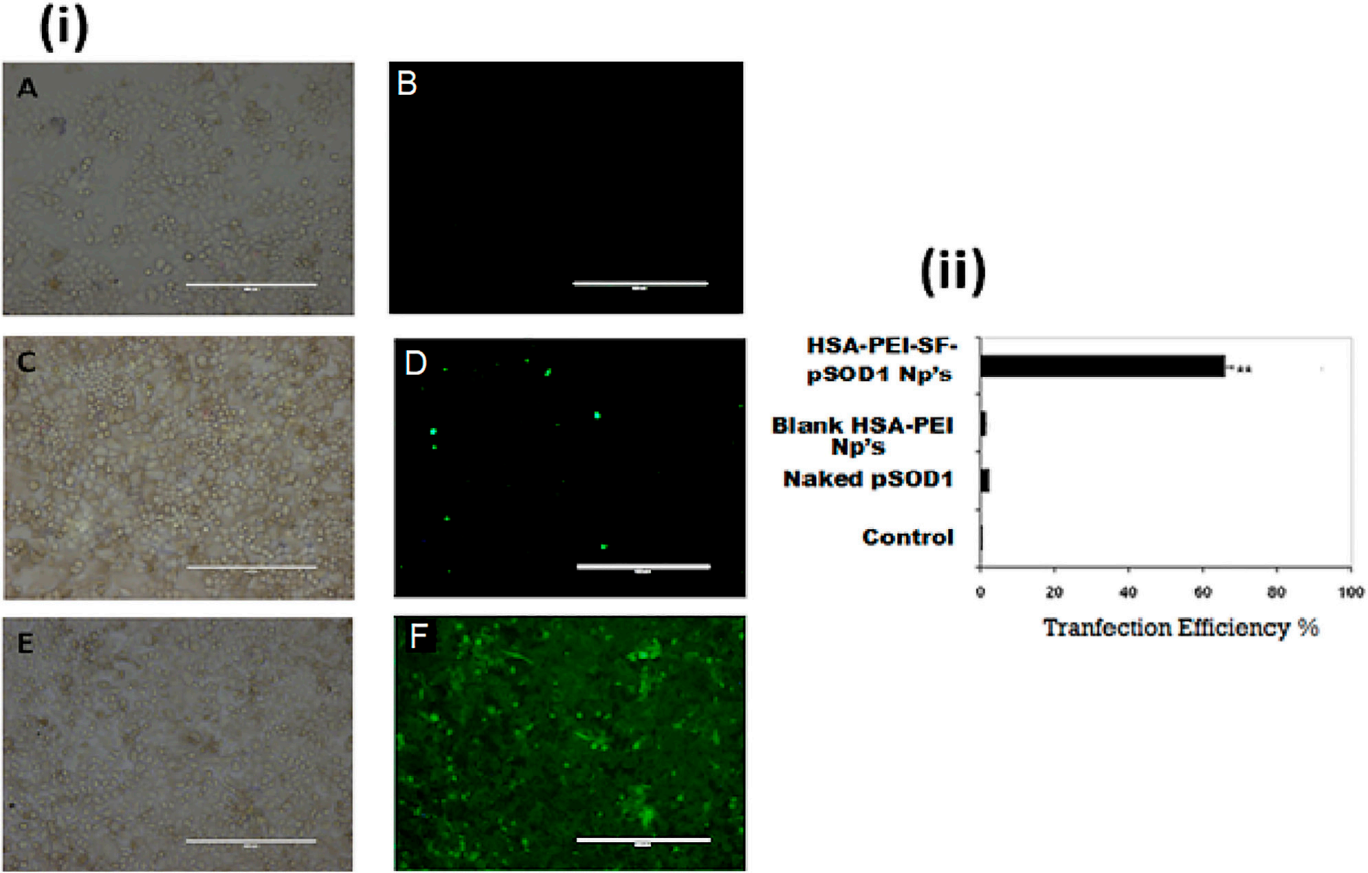
The HSA nanoparticles allow efficient *in vitro* gene transfection and expression of pSOD1 in L-132 cells. (i) **(A,B)**: *in vitro* transfection efficiency with blank HSA-PEI Np’s only; **(C,D)**: transfected with 5000 ng pSOD1 only; **(E,F)**: HSA-PEI-SF-pSOD1 Np’s containing 460 ng pSOD1 following 6 h incubation; transfection was assessed at 48 h post-transfection. Data are expressed as mean ± SD for *n* = 3. ** Indicates a significant difference from the control group at *p* ≤0.01.

We demonstrated the *in vitro* transfection efficiency of pSOD1-SF loaded PEI-HSA Np’s by incubating human lung cancer cells (L-132) with blank HSA-PEI Np’s, naked plasmid pSOD-1, and HSA-PEI-SF-pSOD1 Np’s for 6 h, in serum-free medium followed by 48 h incubation in complete medium. Figure 5 illustrates negligible transfection efficiency for naked plasmid at a pSOD1 dose of 5000 ng, suggesting that the transfection potential of plasmid alone is insignificant. The results signify that blank HSA-PEI nanoparticles also showed 1–2% fluorescence, probably due to the autofluorescence of human serum albumin nanoparticles cross-linked with glutaraldehyde, whereas only 2–3% transfection efficiency was obtained with naked pSOD-1, which is insignificant as compared to the pSOD1-SF loaded PEI-HSA Np’s’ transfection efficiency. The developed NCs demonstrated, on the other hand, HSA-PEI-SF-pSOD1 Np’s results in 66% transfection efficiency, containing 460 ng pSOD1, were indicating that the HSA Np’s’ formulation has high transfection efficiency even at very low concentrations. It is reported that the HSA nanoparticles with the unmodified surface have lower transfection efficiency due to the presence of their negative surface charge, even the negatively charged HSA nanoparticles could not interact efficiently with the negatively charged phosphate backbone of plasmid DNA, and also affects or impedes the cellular uptake of nanoparticles (Torrano et al., 2013). Thus, to overcome these problems, researchers synthesized the cationized HSA nanoparticles surface (Fischer et al., 2001). The HSA nanoparticles with polyethyleneimine (PEI) showed an endosomolytic effect that led to significant gene expression *in vitro* in HEK293 cell lines. Therefore, to enhance cellular uptake and gene transfection efficiency, they did the surface modification with PEG-NH_2_ (Rhaese et al., 2003). In our study, we modified the HSA nanoparticles’ surface with PEI, which exhibits high transfection efficiency i.e. 66% as compared to bare HSA nanoparticles i.e. only 2–3%, this suggested that nanoparticles enter the L-132 cells via the caveolae or clathrin-mediated pathway, showed endosomal escape, and further sustain plasmid release occurs in nucleolus without degradation in the endosomal compartment.

### 3.5 ROS Scavenging Potential of HSA-PEI-SF-pSOD1 Np’s

**FIGURE 6:**
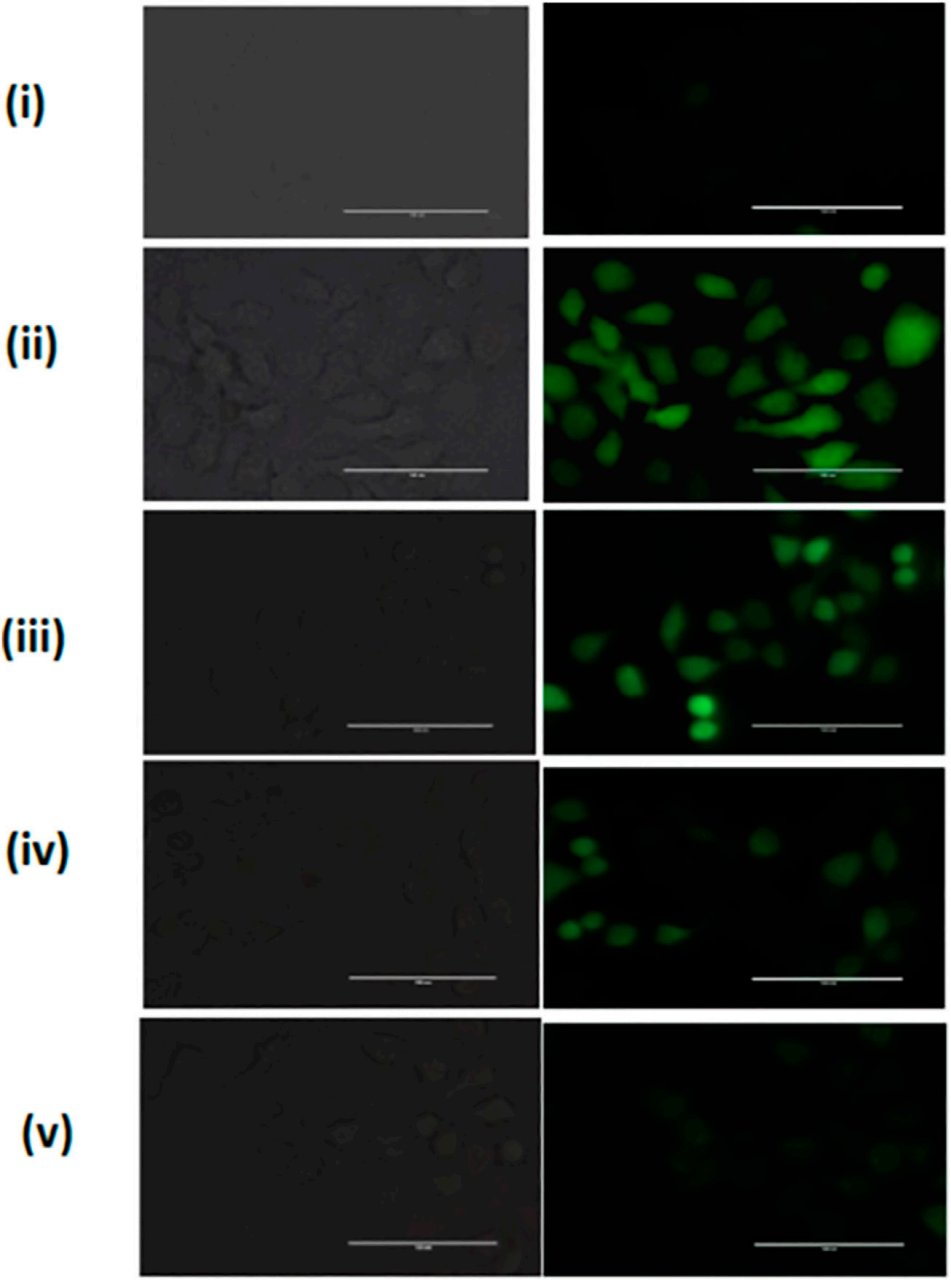
The ROS scavenging in L-132 cells. A Representative fluorescence image was acquired for H_2_O_2_-treated L-132 cells after DCFH-DA dye staining (i) Untreated cells, (ii) H_2_O_2_ treated cells without HSA-PEI-SF-pSOD1 Np’s preincubation, and (iii–v) H_2_O_2_ treated cells with an increase in HSA-PEI-SF-pSOD1 Np’s at 12, 24, and 96 h preincubation time. All the scale bars represent 100 µM.

The ROS scavenging potential of HSA-PEI-SF-pSOD1 Np’s was further assessed in L-132 cells where we have used hydrogen peroxide as positive control which mediates oxidative stress in cells. As depicted in Figure 6, when cells were exposed to HSA-PEI-SF-pSOD1 Np’s before H_2_O_2_ exposure, they were not exhibiting high reactive oxygen species (ROS). Whereas, in Figure 6, we have noticed considerable changes in the level of ROS species at longer incubation time as compared to the initial hours of incubation of nanoparticles. After 96 h the intensity of DCF decreases, this is ascribed may be due to drug internalized inside the cytosolic compartment, and the subsequent expression of SOD1 transgene occurs in 96 h. On the other hand, L-132 cells exhibit high DCF fluorescence intensity, where H_2_O_2_ exposure was given without HSA-PEI-SF-pSOD1 Np’s preincubation, suggesting removal of ROS species by nanoparticles was successfully achieved.

## 4 Conclusion

We have developed HSA-PEI-SF-pSOD1 nanocomposites by using the desolvation cross-linking method to fabricate PEI-stabilized HSA nanoparticles that have been loaded with SF drug and pSOD1 plasmid. Here, PEI acts as a nonviral vector and because of its positive charge of PEI, it condenses the DNA and is endocytosed by the negative-charged cell membrane. Therefore, PEI conjugated HSA nanoparticles show potential for developing into an effective carrier for various drugs. This approach can also be tried for treating neuronal diseases which have a scope to treat with gene delivery. Genetic treatment has a scope in the near future where such carriers will play a very crucial role. Moreover, these carriers can deliver the therapeutic material to the desired site of action with relevant concentration, which further lessens the side effects associated with the drug. Antioxidant and cytoprotective effects of HSA-PEI-SF-pSOD1 nanoformulations have been examined *in vitro* against human lung epithelial cells (L-132). The MTT assay has shown high biocompatibility of approx. >95% up to 96 h, whereas the time-dependent SOD activity study has demonstrated high antioxidant activity of nanoformulations. The expression of GFP-tagged transgene SOD1 has been examined by a fluorescence microscope and revealed the transfection efficiency of around 66% in L-132 transfected cells. In summary, our results have demonstrated that HSA-PEI-SF-pSOD1 nanoformulations with a loading dose of SF 3 µg and 460 ng pSOD-1 ensure a combined therapeutic effect owing to the initial release of the SF drug followed by expression of the SOD 1 gene. This provides reference data for both *in vitro* and *in vivo* studies for the future.

## Data Availability

The raw data supporting the conclusions of this article will be made available by the authors, without undue reservation.
