# Supplementary material for: Albumin-Based Nanocarriers for the Simultaneous Delivery of Antioxidant Gene and Phytochemical to Combat Oxidative Stress
Source: Front Cell Dev Biol. 2022 Aug 12;10:846175. doi: 10.3389/fcell.2022.846175 (PMC9412823; doi:10.3389/fcell.2022.846175)
Supplement: Supplementary file 1 [file DataSheet1.pdf]

Table 1. Average DLS diameter, polydispersity index (PdI) and zeta potential of the HSA nanoparticles.

Sample	Average diameter (nm)	PdI	Zeta potential (mV)
HSA NPs	174.5	0.144	-6.4
HSA-PEI NPs	267.5	0.081	59.5
HSA-SF NPs	494.4	0.213	-16.2
HSA-PEI-SF NPs	522.1	0.176	57.4
HSA-pSOD1 NPs	385.4	0.161	-15.3
HSA-PEI-pSOD1 NPs	468.2	0.134	50.9
HSA-SF-pSOD1 NPs	521.8	0.098	-18.2
HSA-PEI-SF-pSOD1 NPs	668.6	0.225	53.8
